# Systems pharmacology dissection of pharmacological mechanisms of Xiaochaihu decoction against human coronavirus

**DOI:** 10.1186/s12906-023-04024-6

**Published:** 2023-07-20

**Authors:** Lvjie Xu, Chuipu Cai, Jiansong Fang, Qihui Wu, Jun Zhao, Zhe Wang, Pengfei Guo, Lishu Zheng, Ailin Liu

**Affiliations:** 1 State Key Laboratory of Bioactive Substances and Functions of Natural Medicines, Institute of Materia Medica, Chinese Academy of Medical Sciences and Peking Union Medical College, Beijing, 100050 China; 2 Beijing Key Laboratory of Drug Target Identification and Drug Screening, Institute of Materia Medica, Chinese Academy of Medical Sciences and Peking Union Medical College, Beijing, 100050 China; 3 Department of Pharmacy, Beijing Tongren Hospital, Capital Medical University, Beijing, 100730 China; 4 Division of Data Intelligence, Department of Computer Science, Key Laboratory of Intelligent Manufacturing Technology of Ministry of Education, Shantou University, Shantou, China; 5 Science and Technology Innovation Center, Guangzhou University of Chinese Medicine, Guangzhou, China; 6 NHC Key Laboratory of Medical Virology and Viral Diseases, National Institute for Viral Disease Control and Prevention, China CDC, Beijing, China; 7 Center for Biosafety Mega-Science, Chinese Academy of Sciences, Beijing, China

**Keywords:** Xiaochaihu decoction, Human coronavirus, Systems pharmacology, Molecular mechanism, HCoV-229E virus

## Abstract

**Background:**

Although coronavirus disease 2019 (COVID-19) pandemic is still rage worldwide, there are still very limited treatments for human coronaviruses (HCoVs) infections. Xiaochahu decoction (XCHD), which is one of the traditional Chinese medicine (TCM) prescriptions in Qingfeipaidu decoction (QFPDD), is widely used for COVID-19 treatment in China and able to relieve the symptoms of fever, fatigue, anorexia, and sore throat. To explore the role and mechanisms of XCHD against HCoVs, we presented an integrated systems pharmacology framework in this study.

**Methods:**

We constructed a global herb-compound-target (H-C-T) network of XCHD against HCoVs. Multi-level systems pharmacology analyses were conducted to highlight the key XCHD-regulated proteins, and reveal multiple HCoVs relevant biological functions affected by XCHD. We further utilized network-based prediction, drug-likeness analysis, combining with literature investigations to uncover the key ani-HCoV constituents in XCHD, whose effects on anit-HCoV-229E virus were validated using cytopathic effect (CPE) assay. Finally, we proposed potential molecular mechanisms of these compounds against HCoVs via subnetwork analysis.

**Results:**

Based on the systems pharmacology framework, we identified 161 XCHD-derived compounds interacting with 37 HCoV-associated proteins. An integrated pathway analysis revealed that the mechanism of XCHD against HCoVs is related to TLR signaling pathway, RIG-I-like receptor signaling pathway, cytoplasmic DNA sensing pathway, and IL-6/STAT3 pro-inflammatory signaling pathway. Five compounds from XCHD, including betulinic acid, chrysin, isoliquiritigenin, schisandrin B, and (20R)-Ginsenoside Rh1 exerted inhibitory activity against HCoV-229E virus in Huh7 cells using in vitro CPE assay.

**Conclusion:**

Our work presented a comprehensive systems pharmacology approach to identify the effective molecules and explore the molecular mechanism of XCHD against HCoVs.

**Supplementary Information:**

The online version contains supplementary material available at 10.1186/s12906-023-04024-6.

## Introduction

Coronavirus (CoV) is a kind of single-stranded RNA virus that can infect many animal species which can lead to different degrees of lesions in the respiratory tract, liver, intestines and nervous system [1]. CoV contains a total of 4 subfamilies, including α-CoV, β-CoV, γ-CoV and δ-CoV, of which α and β subfamilies are capable of infecting mammals including humans [2]. At present, the human coronaviruses (HCoVs) include HCoV-OC43 (β-CoV) [3], HCoV-229E (α-CoV) [4], HCoV-NL63 (α-CoV) [5], and HCoV-HKU1 (β-CoV) [6]. In the past 20 years, three highly pathogenic CoVs prevailed worldwide, which are severe acute respiratory syndrome coronavirus (SARS-CoV), Middle East respiratory syndrome coronavirus (MERS-CoV) and severe acute respiratory syndrome coronavirus 2 (SARS-CoV-2) [7]. As of March 10, 2023, the number of confirmed cases of the coronavirus disease 2019 (COVID-19) has reached up to 651 million with a cumulative death of exceeded 6.8 million globally [8] (https://coronavirus.jhu.edu/map.html). Thus, it is of great urgency to develop the new effective anti-HCoVs drugs for patient therapy.

Preliminary clinical practice evidences showed that traditional Chinese medicine (TCM) prescriptions, especially those consisting of a combination of herbs, have achieved beneficial effect for COVID-19 patients by shortening of hospitalization duration and reducing the chance of getting complications as well as the mortality rate [9, 10]. Qingfeipaidu decoction (QFPDD) consisted of four classical TCM prescriptions [11], is currently used as a common prescription in the *COVID-19 diagnosis and treatment scheme* in China. From a retrospective multicenter study on 782 COVID-19 patients from 54 hospitals in nine provinces of China, compared with treatment with QFPDD initiated after 3 weeks of infection, treatment started less than 1 week, 1–2 weeks, or 2–3 weeks had a significantly shorter recovery time, with adjusted hazard ratio of 3.81 (2.65–5.48), 2.63 (1.86–3.73), and 1.92 (1.34–2.75), respectively [12]. As one of the four prescriptions in QFPDD, Xiaochaihu Decoction (XCHD) plays the roles of relieving symptoms of fever, fatigue, anorexia and sore throat after SARS-CoV-2 infection [13]. Clinical observation indicated that modified XCHD exhibited beneficial effects on COVID-19 patients, with a response rate of 96.43% after 1 to 2 weeks of treatment. Early XCHD treatment can completely relieve the condition of patients with mild and moderate symptoms, and prevent them progressing into severe stage [14]. XCHD is composed of seven medicinal herbs, which are *Bupleurum chinense* DC. (Chaihu), *Scutellaria baicalensis* Georgi. (Huangqin), *Pinellia ternata* (Thunb.) Breit.(Banxia), *Zingiber officinale* Roscoe (Shengjiang), *Panax ginseng* C.A.Mey. (Renshen), *Glycyrrhiza uralensis* Fisch. (Zhigancao) and *Ziziphus jujuba* Mill. (Dazao). Previous pharmacological studies showed the effects of XCHD on resisting various RNA and DNA viruses (including coronaviruses) infection, improving immune function and restoring body temperature by hypothalamus regulating [15, 16]. Chaihu has been demonstrated to curb the infections of influenza virus, hepatitis virus, and other viruses [17, 18]. Huangqin, was proved to enhance leukocyte phagocytosis, increase free antibodies, inhibit the release of active substances and regulate immune function [19, 20]. Although the pharmacological actions of XCHD make it a treatment option for COVID-19, there is a great challenge to evaluate the efficacy, identify the functional constituents contained, and explore its molecular mechanisms against COVID-19.

TCM is characterized by complex components, multiple targets and synergistic actions. Systems pharmacology had been recentely developed in TMC studies, it provided a network perspective to explore the relationship between components and targets [21]. It has been shown that systems pharmacology gave an effective approaches to identify the bioactive compounds, predict the corresponding targets and elucidate the molecular mechanisms about how does TCM work on different diseases [22, 23]. For instance, by using this strategy, it uncovered the mechanism of Huanglian-Wuzhuyu herb pair in treating nonalcoholic steatohepatitis and predicted active ingredients, it also explained mechanism of Lianhuaqingwen capsule in treating COVID-19 [24, 25].

**Fig. 1:**
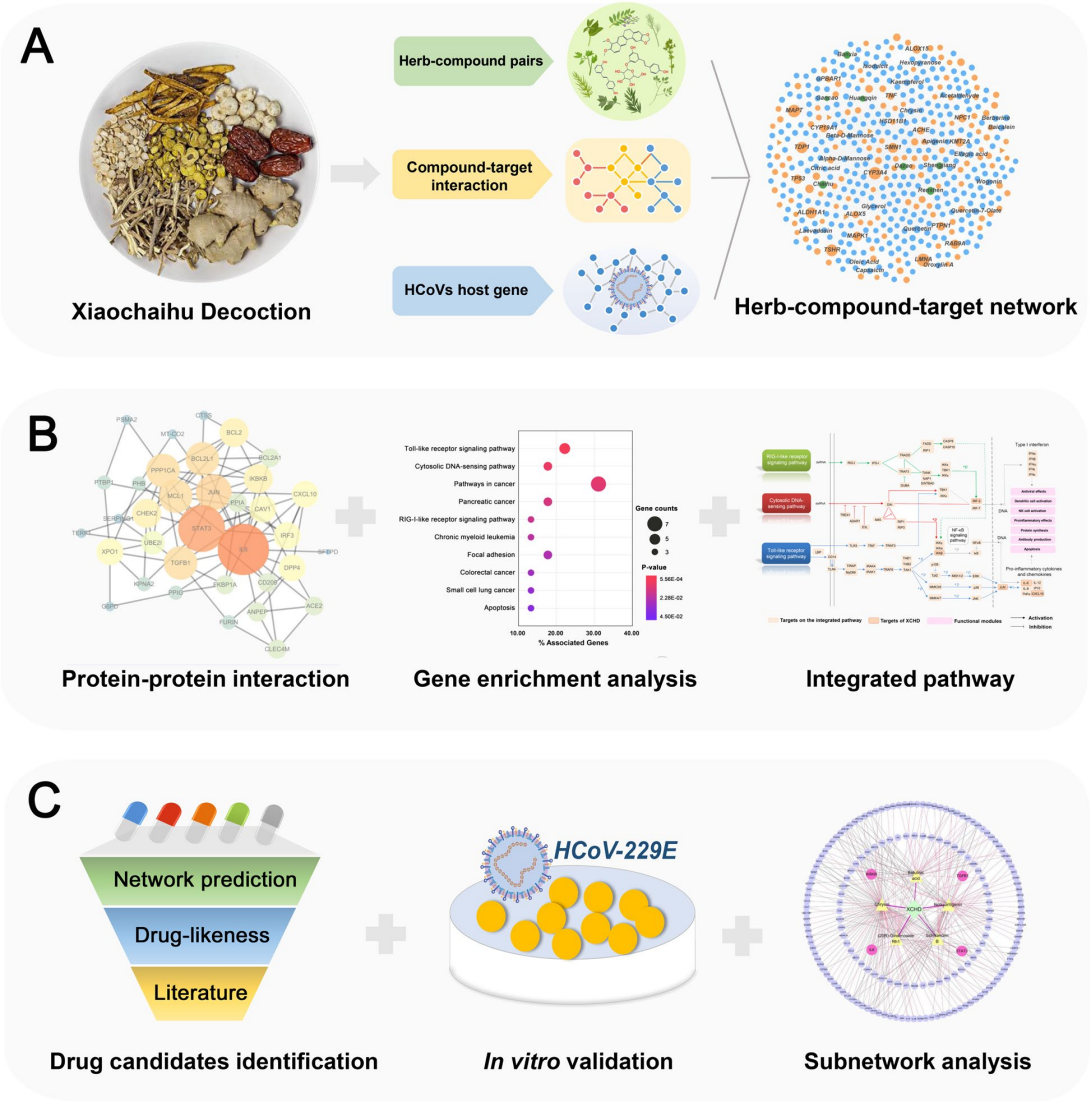
Schematic of the systems pharmacology infrastructure for uncovering the molecular mechanism of XCHD against HCoVs

In this study, we presented a systems pharmacology-based framework to identify the effective components of XCHD and explore the underlying mechanism of XCHD against HCoVs (Fig. 1). Briefly, we first constructed a global herb-compound-target (H-C-T) network for XCHD, which integrated compounds from XCHD, known targets from published experimental literatures, and computationally putative targets predicted from balanced substructure-drug-target network-based inference (bSDTNBI) method [26, 27]. Subsequently, a gene set of HCoVs-associated proteins was mapped into the H-C-T network to determine the HCoVs-associated targets that can be regulated by XCHD. Then we applied specific anti-HCoV compound-target (C-T) network, protein–protein interaction (PPI) network, gene set enrichment, and integrated pathway analysis to comprehensively explore the potential biological functions and signaling pathways affected by XCHD. Furthermore, in silico strategy was utilized to identify the active anti-HCoVs components in XCHD by integrating network-based analysis and drug-likeness prediction. Finally, in vitro HCoV-229E virus-induced cytopathic effect assay was carried out to validate the anti-HCoV activity of predicted compounds, while subnetwork analysis was used to investigate the specific synergistic mechanisms of the main active compound candidates in XCHD.

## Materials and methods

### Compounds and virus

XCHD is composed of seven medicinal herbs, including Chaihu, Huangqin, Banxia, Shengjiang, Renshen, Zhigancao and Dazao. The scientific species names of each herb are shown in Table 1.

**Table 1:** The scientific species names of herbs in XCHD

Herb	Latin Binomial Name
Chaihu	*Bupleurum chinensis* DC
Huangqin	*Scutellaria baicalensis* Georgi
Zhigancao	*Glycyrrhiza uralensis* Fisch
Banxia	*Pinellia ternata* (Thunb.) Breit
Renshen	*Panax ginseng* C. A. Meyer
Shengjiang	*Zingiber officinale* Roscoe
Dazao	*Ziziphus jujuba* Mill

Total 30 compounds from these herbs for in vitro assays were commercially obtained from Topscience Biochemical Technology Co., Ltd. The quality control assessment was performed by NMR or HPLC–MS to assure the purities are all greater than 95%.

Huh7 cells and HCoV-229E were kindly provided from CAMS Key Laboratory of Antiviral Drug Research, Institute of Medicinal Biotechnology, Chinese Academy of Medical Sciences and Peking Union Medical College.

### Collection of herbal compounds in XCHD

The chemical structures of all compounds in XCHD were collected from the following databases: Traditional Chinese medicine integrative database (TCMID) [28], TCM Database@Taiwan [29], Traditional Chinese medicine systems pharmacology database and analysis platform (TCMSP) [30], Database of Traditional Chinese Medicine on Immuno-Oncology (TCMIO) [31], and the database and analytical system for network pharmacology analysis for TCM preparations (TCM-MESH) [32]. All compounds were converted to InChIKey and SMILES formats by Open Babel (version 2.3.2) [33]. Compounds with identical structures were merged. The above databases were searched until April 2020.

### Target identification of compounds in XCHD

Both known and predicated targets of XCHD were included in the current study. The known targets were extracted from the previous integrated database [26], which contains 7,030 experimentally validated compound-target interactions (CTIs) collected from ChEMBL (v21) and Binding DB. The predictive network model was established by bSDTNBI method to prioritize potential targets for natural products by resource-diffusion processes of the substructure-drug-target network [26]. The tunable parameters α (initial resource allocation of different node types), β (weighted values of different edge types), γ (influence of hub nodes) and k (number of resource-diffusion processes) were set as 0.1, 0.1, -0.5, and 2, respectively. The substructure items of each compound were calculated using molecular fingerprint Klekota − Roth from PaDEL-Descriptor (version 2.18) [34].

### Collection of HCoV-associated genes

We comprehensively retrieved the literatures to obtain the human genes associated with multiple types of HCoVs, including HCoV-OC43, HCoV-229E, HCoV-NL63, HCoV-HKU1, SARS-CoV, MERS-CoV and SARS-CoV-2. The names of the collected genes were standardized into gene symbol according to GeneCards [35] (retrieved until Jan 2021) and UniProt [35, 36] (retrieved until Jan 2021), while the duplicates were removed.

### Drug-likeness screening

In this study, drug-likeness analysis was conducted using a classification model based on random forest (RF) method, the analysis was available at ADMETlab platform (https://admetmesh.scbdd.com/) and completed in April 2021. Specifically, the RF model was trained by 6,731 positive samples from DrugBank and 6,769 negative samples from ChEMBL with IC_50_ or Ki values < 10 μm. The obtained model has good ability to generalize the new chemical entity with classification accuracy of 0.800 and AUC score of 0.867 on external test set. More detailed information can be found in previous study [37].

### Network construction

In our study, we generated three types of networks to explore the molecular mechanism of XCHD against HCoVs, including compound-target (C-T) networks, target-pathway network and protein–protein interaction (PPI) network. These networks were constructed by Gephi (v0.9.2, https://gephi.org/) and Cytoscape (v3.2.1, http://www.cytoscape.org/). The compounds, pathways and genes (targets) were represented as nodes. The interactions were denoted as edges. The degree of each node was defined by the number of edges linked to it, which represents the hierachy of the node in the network. For the PPI network, the functional relationships among interacting proteins were generated through STRING database [38]. The protein type was defined as "*Homo* Sapiens", while the reliability score of the PPI edge required interaction score was set as greater than 0.4.

### Gene enrichment analysis

To explore how do the targets of XCHD exert their anti-HCoVs effect through pathway regulation, we annotated the biological functions of the HCoVs targets of XCHD to find out the potential signaling pathways and biological processes. Gene ontology (GO) term and KEGG pathway enrichment analysis (https://www.kegg.jp/kegg/kegg1.html) [39] were performed by The Database for Annotation, Visualization and Integrated Discovery (DAVID 6.8 database, https://david.ncifcrf.gov/) [40] (retrieved until Apr 2021). The biological processes, molecular functions, cellular components and signaling pathways with *p* < 0.05 were considered as statistically significantly enriched.

### Cytotoxicity test and cytopathic effect (CPE) reduction assay

Huh7 cells seeded in 96-well culture plates were incubated in a 37 °C and humidified 5% CO_2_ atmosphere until reaching 80% confluency. For cytotoxicity test, the untreated cells were used as a reference; other cells were treated with the test drugs of 8 serially diluted concentrations.

For CPE assay, the cells were either infected with 100 TCID_50_ HCoV-229E only or both treated with 100 TCID_50_ HCoV-229E and the different drugs. Ribavirin treatment was used as the positive control for HCoV-229E infection inhibition. The cells were incubated until the CPE of viral wells reached 4 + (0 means no CPE; 1 + means CPE is 1%-25%; 2 + means CPE is 26%-50%; 3 + means CPE is 51%-75%; 4 + means CPE is 76%-100%). The inhibition rates of each drug on HCoV-229E in Huh7 cells were calculated by normalizing the CPE of each group to the CPE of virus-only well.

The half toxic concentration (TC_50_) and the half inhibitory concentration (IC_50_) of each test sample were calculated using the Reed-Muench method.

### LibDock operation

Molecular docking is a process that identifies the complementary molecules for a target spatially and electrically. The target protein structures were downloaded through the PDB protein database (https://www.rcsb.org) [41], and then imported into Discovery Studio 2016 software with small molecule structures. After protein and small molecule structure modification, LibDock was operated for molecular docking, with LibDock scores calculated as the assessment of molecular conformational affinity.

## Results

### Analysis of HCoV-associated targets of XCHD

Previous preclinical studies and clinical trials demonstrated that the multi-component synergy of TCM are related to the interaction between components [42, 43]. To analyze the chemical composition and pharmacodynamic material basis of XCHD, a total of 1,899 compounds in XCHD were obtained after removing the duplicates with identical chemical structures (Supplementary Table S1). The numbers of ingredients of each herb in XCHD are 538 (Chaihu), 175 (Huangqin), 270 (Banxia), 472 (Shengjiang), 627 (Renshen), 18 (Zhigancao), and 253 (Dazao), respectively.

**Fig. 2:**
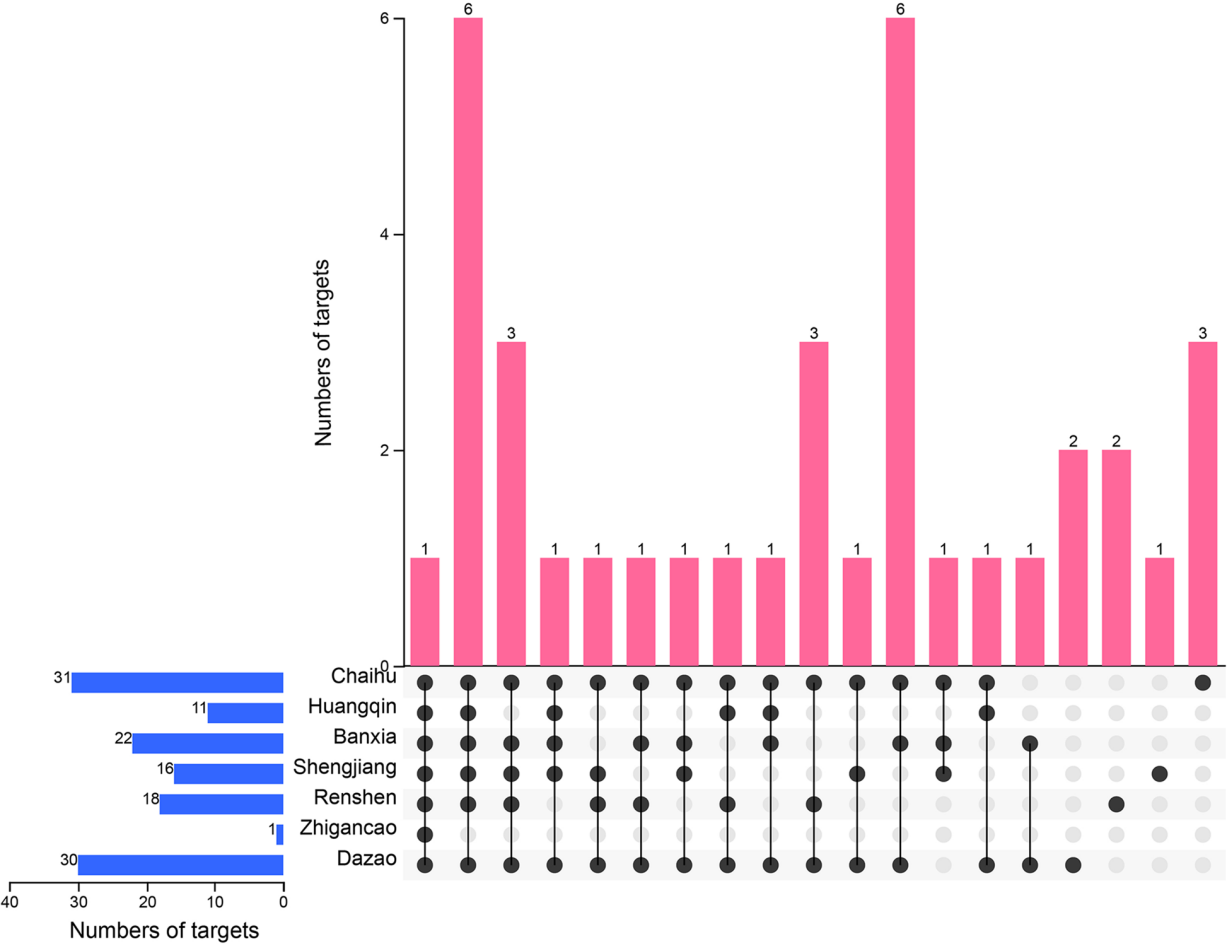
HCoV-associated target distribution of various herbs in XCHD. Blue bars represent the number of targets for each herb, red bars represent the number of targets covered by single or multiple herbs, the dots indicate the targets associated by the ingredients in the corresponding herbs

To understand the potential synergistic effect mechanism of the herbs against HCoV, we used UpSet Wayne diagram to analyze the distribution of HCoV-associated genes regulated by XCHD (Fig. 2). There are a total of 37 HCov-associated targets regulated by all 7 herbs. In specific, the numbers of HCoV-associated genes targeted by the components of Chaihu, Huangqin, Banxia, Shengjiang, Renshen, Zhigancao and Dazao are 31, 11, 22, 16, 18, 1 and 30, respectively. Interestingly, inhibitor of kappa light polypeptide gene enhancer in B-cells, kinase beta (IKBKB) was the only common gene targeted by all seven herbs (Table 2), suggesting that IKBKB might be the most key target for the synergistic effect. In addition, we also observed there are 6 genes targeted by 6 herbs and 4 genes targeted by 5 herbs. The targets distribution reflects the potential mechanism of how XCHD performs synergistic effect against HCoVs.

**Table 2:** Herbs in XCHD and their corresponding HCoV-associated host targets

Herb	Target gene symbol
Chaihu	ACE2, ANPEP, BCL2, BCL2A1, BCL2L1, CAV1, CD209, CHEK2, CLEC4M, CTSS, CXCL10, DPP4, FKBP1A, FURIN, G6PD, GBF1, HGS, IKBKB, IL6, IRF3, JUN, KPNA2, MCL1, PPIA, PPP1CA, PTBP1, SERPING1, SFTPD, STAT3, TGFB1, XPO1
Huangqin	ACE2, BCL2, BCL2L1, CAV1, CHEK2, IKBKB, IL6, JUN, MCL1, STAT3, TGFB1
Banxia	ANPEP, BCL2, BCL2A1, BCL2L1, CHEK2, DPP4, FURIN, G6PD, GBF1, HGS, IKBKB, IL6, IRF3, JUN, KPNA2, MCL1, PPIA, PPIG, PPP1CA, PTBP1, STAT3, TGFB1
Shengjiang	BCL2, BCL2L1, CXCL10, DPP4, FKBP1A, FURIN, G6PD, IKBKB, IL6, IRF3, JUN, MCL1, PPIA, PSMA2, STAT3, TGFB1
Renshen	ACE2, ANPEP, BCL2, CD209, CLEC4M, DPP4, FKBP1A, FURIN, IKBKB, IL6, JUN, MCL1, PPIA, SFTPD, STAT3, TERF1, TGFB1, UBE2I
Zhigancao	IKBKB
Dazao	ACE2, ANPEP, BCL2, BCL2A1, BCL2L1, CAV1, CD209, CHEK2, CLEC4M, COX2, CXCL10, DPP4, FKBP1A, FURIN, GBF1, HGS, IKBKB, IL6, IRF3, JUN, KPNA2, MCL1, PHB, PPIA, PPIG, PPP1CA, PTBP1, SFTPD, STAT3, TGFB1

### Network construction and mechanisms analysis of XCHD against HCoVs

**Fig. 3:**
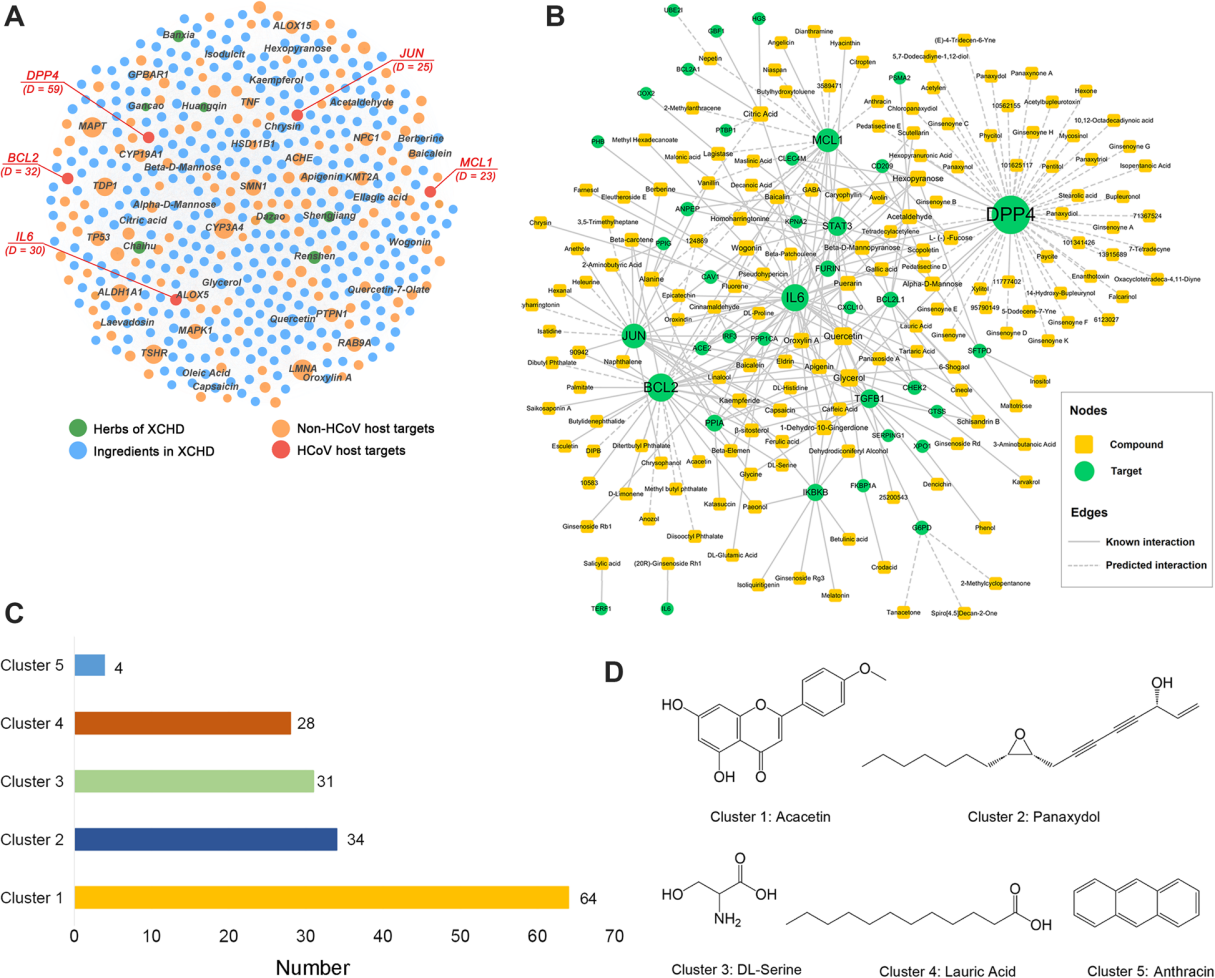
Network construction of XCHD against HCoVs. **A** A global herb-compound-target (H-C-T) network for XCHD. For demonstration purposes, only nodes with degree larger than 20 are displayed. The labels of the top 20 targets and compounds with highest degrees are displayed. **B** A specific compound-target (C-T) network of XCHD against HCoVs. The node size is proportional to degree. Chemical scaffold clustering analysis of the 161 XCHD constituents targeting to HCoV-associated genes (**C**) and the center chemical structures (**D**)

In the study, to identify the effective components of XCHD against HCoV, we performed systems pharmacology-based framework analysis and obtained 344 components in XCHD connecting to 2,656 known targets and 561 predicated targets (Supplementary Table S2 and Table S3). We thus obtained 2,823 potential protein targets of XCHD by merging the known and predicted ones (Supplementary Table S4). Furthermore, a global H-C-T network was constructed by integrating herb-compound pairs and compound-target interactions (CTIs), which is composed of 4,729 nodes (7 herbs, 1,899 compounds, and 2,823 protein targets) and 47,587 edges (24,545 herb-compound pairs and 45,133 CTIs). As illustrated in Fig. 3A, compounds and targets with degrees (*D*) larger than 20 were displayed. Most compounds were connected to multiple shared targets. Of note, the H-C-T network also indicated that several important HCoVs associated genes with high degrees (*D* > 20), including DPP4 (*D* = 59), BCL2 (*D* = 32), IL6 (*D* = 30), JUN (*D* = 25) and MCL1 (*D* = 23). Overall, it is likely that the ingredients in XCHD work to prevent HCoVs infection by regulating multiple HCoV-associated targets.

In our study, 90 HCoV-associated targets were extracted from pharmacological references (Supplementary Table S5). These genes were mapped into the H-C-T network to explore the relationship between XCHD and HCoVs infection. To do this, we extracted the HCoVs-specific CTIs from the H-C-T network to build a C-T network for further exploring the potential anti-HCoVs ingredients of XCHD and their corresponding targets (Supplementary Table S6). Our analysis reveled that 161 compounds were connected to 37 HCoV-associated targets in the network (Fig. 3B and Table 3). Among these interactions, 12 compounds interacted with more than 4 targets and 8 targets were linked to more than 10 compounds. The average connectivity of each target and compounds in the network is 7.62 and 1.73, respectively. These results suggested the potential candidate compounds and therapeutic targets of XCHD for its anti-HCoV effects.

**Table 3:** List of the 37 potential HCoVs host targets of XCHD

Target gene symbol	Full name
MCL1	BCL2 family apoptosis regulator
BCL2L1	BCL2 like 1
BCL2A1	BCL2 related protein A1
BCL2	BCL2, apoptosis regulator
CXCL10	C-X-C motif chemokine ligand 10
CLEC4M	C-type lectin domain family 4 member M
CD209	CD209 molecule
FKBP1A	FK506 binding protein 1A
JUN	Jun proto-oncogene, AP-1 transcription factor subunit
ANPEP	alanyl aminopeptidase, membrane
ACE2	angiotensin I converting enzyme 2
CTSS	cathepsin S
CAV1	caveolin 1
CHEK2	checkpoint kinase 2
COX2	cytochrome coxidase subunit II
DPP4	dipeptidyl peptidase 4
XPO1	exportin 1
FURIN	furin, paired basic amino acid cleaving enzyme
G6PD	glucose-6-phosphate dehydrogenase
GBF1	golgi brefeldin A resistant guanine nucleotide exchange factor 1
HGS	hepatocyte growth factor-regulated tyrosine kinase substrate
IKBKB	inhibitor of kappa light polypeptide gene enhancer in B-cells, kinase beta
IRF3	interferon regulatory factor 3
IL6	interleukin 6
KPNA2	karyopherin subunit alpha 2
PPIA	peptidylprolyl isomerase A
PPIG	peptidylprolyl isomerase G
PTBP1	polypyrimidine tract binding protein 1
PHB	prohibitin
PSMA2	proteasome subunit alpha 2
PPP1CA	protein phosphatase 1 catalytic subunit alpha
SERPING1	serpin family G member 1
STAT3	signal transducer and activator of transcription 3
SFTPD	surfactant protein D
TERF1	telomeric repeat binding factor 1
TGFB1	transforming growth factor beta 1
UBE2I	ubiquitin conjugating enzyme E2 I

The chemical scaffold clustering analysis of 161 XCHD constituents regulating to HCoV-associated genes was operated by Discovery Studio. The chemical fingerprint of FCFP6 was used and compounds with similar Tanimoto distance were clustered together. These compounds were clustered into five groups, with the number of compounds in cluster 1 to 5 is is 64, 34, 31, 28 and 4, respectively (Fig. 3C). The structures of each cluster center are acacetin, panaxydol, DL-serine, lauric acid and anthracin, respectively (Fig. 3D).

### Protein–protein interaction (PPI) network uncovering the core anti-HCoVs targets of XCHD

**Fig. 4:**
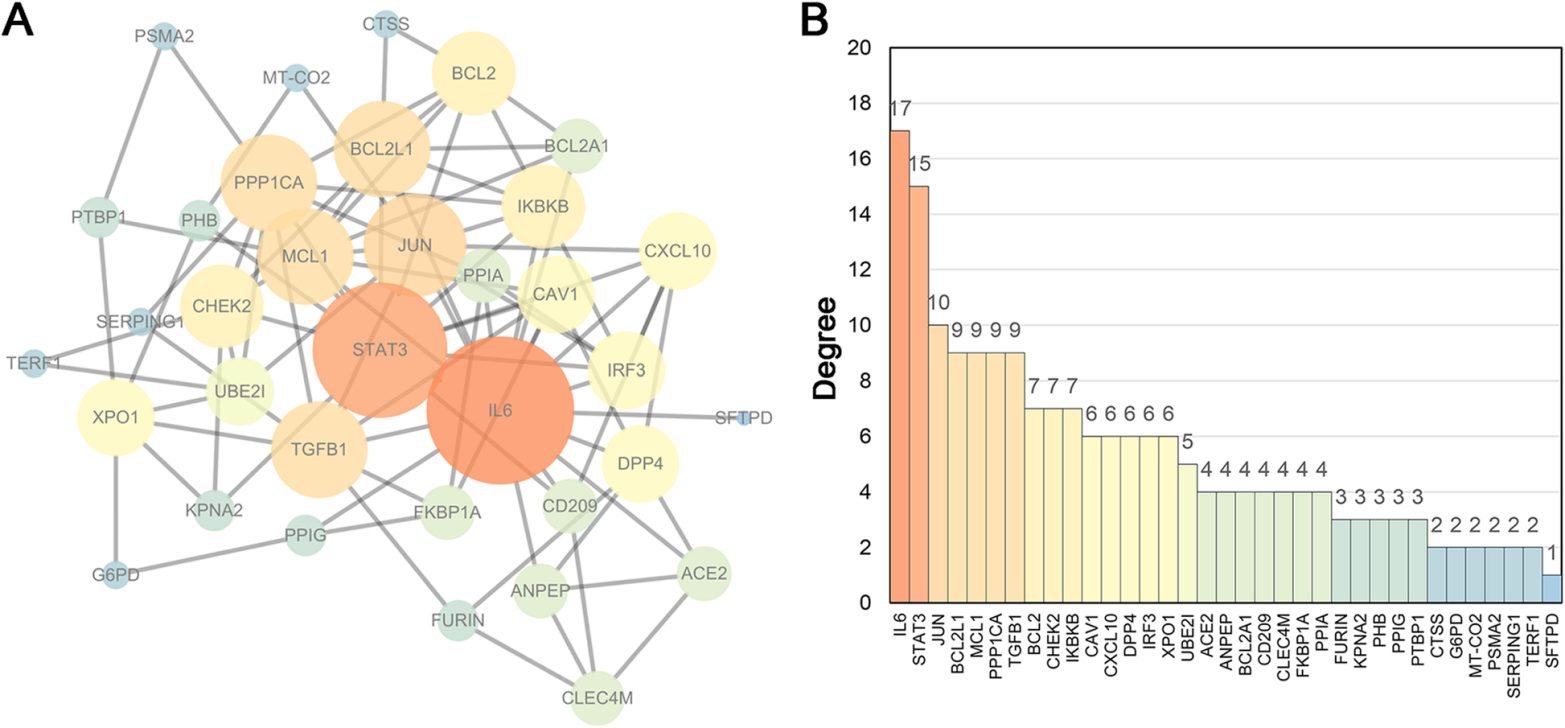
Protein–protein interaction (PPI) of HCoVs-associated targets acted by XCHD. **A** PPI network. Nodes represent targets and edges represent the interactions among targets. The color brightness and the size of nodes are proportional to degree. **B** Degrees of the targets in the PPI network

To explore the proteins anti-HCoV activity, we constructed a PPI network for the 37 HCoV-associated targets regulated by XCHD. Node with larger circular diameter and brighter color indicates more protein interaction pairs. As shown in Fig. 4A, there are 95 protein interaction pairs within all these 35 proteins, and the average interactions number for each protein is 5.43. Among these proteins, IL-6 and STAT3 are the core proteins with degrees of 17 and 15, respectively (Fig. 4B). Considering the key roles of IL6 and STAT3 in immune functions, it makes sence that XCHD could prevent HCoVs infection by promoting inflammatory signals and regulating the innate and adaptive immune responses.

### Gene enrichment analysis of HCoVs-associated host targets of XCHD

**Fig. 5:**
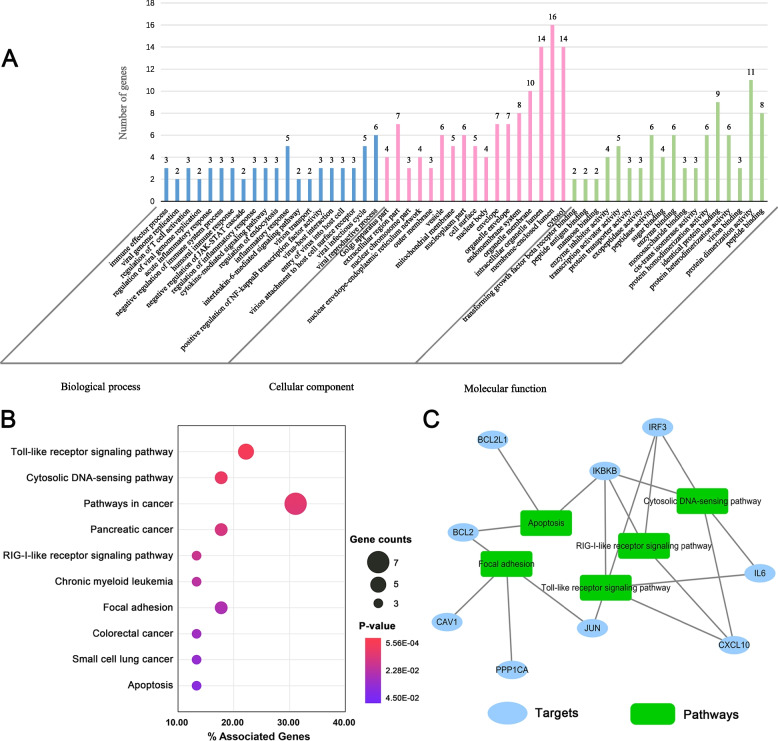
Gene enrichment analysis of the HCoVs-associated host targets of XCHD. **A** GO terms annotation; (**B**) KEGG pathway enrichment; (**C**) Target-pathway network

To explore the molecular mechanism of XCHD against HCoVs, we made GO annotation and KEGG pathway enrichment of the HCoVs-associated host targets. As shown in Fig. 5A, the HCoVs associated targets regulated by XCHD are localized in various compartments of cell, including cell membrane, cytoplasm, mitochondria, Golgi apparatus and cell nucleus, etc. These targets are involved in virus binding, sugars binding, enzymes binding, and transforming growth factor β receptor binding. In addition, their functions cover enzyme inhibition, protein transport, transcription activation, and other activities. Furthermore, they also participate in the activities like the viral cell entry, transport, gene replication, immune response function regulation, and acute inflammation.

We further utilized KEGG pathway enrichment analysis to uncover the biological pathways using of these 37 proteins. As presented in Fig. 5B, the cancer-related pathways, toll-like receptor (TLR) signaling pathway (hsa04620, *p* = 5.56E-04), retinoic acid-inducible gene (RIG)-I-like receptor signaling pathway (hsa04622, *p* = 0.031), cytosolic DNA-sensing pathway (hsa04623, *p* = 0.001), focal adhesion (hsa04510, *p* = 0.042), and apoptosis (hsa04210, *p* = 0.045) were all significantly enriched (*p* < 0.05 was considered as significant). Moreover, we constructed a target-pathway network to illustrate the relationships between these HCoVs-associated targets and the enriched pathways. The network is composed of 19 target-pathway interactions between 9 targets and 5 potential signaling pathways (Fig. 5C). In summary, the gene enrichment analysis indicated that XCHD prevents HcoVs infection by mainly regulating the immune function of host cells.

### Integrated anti-HCoVs pathway of XCHD

**Fig. 6:**
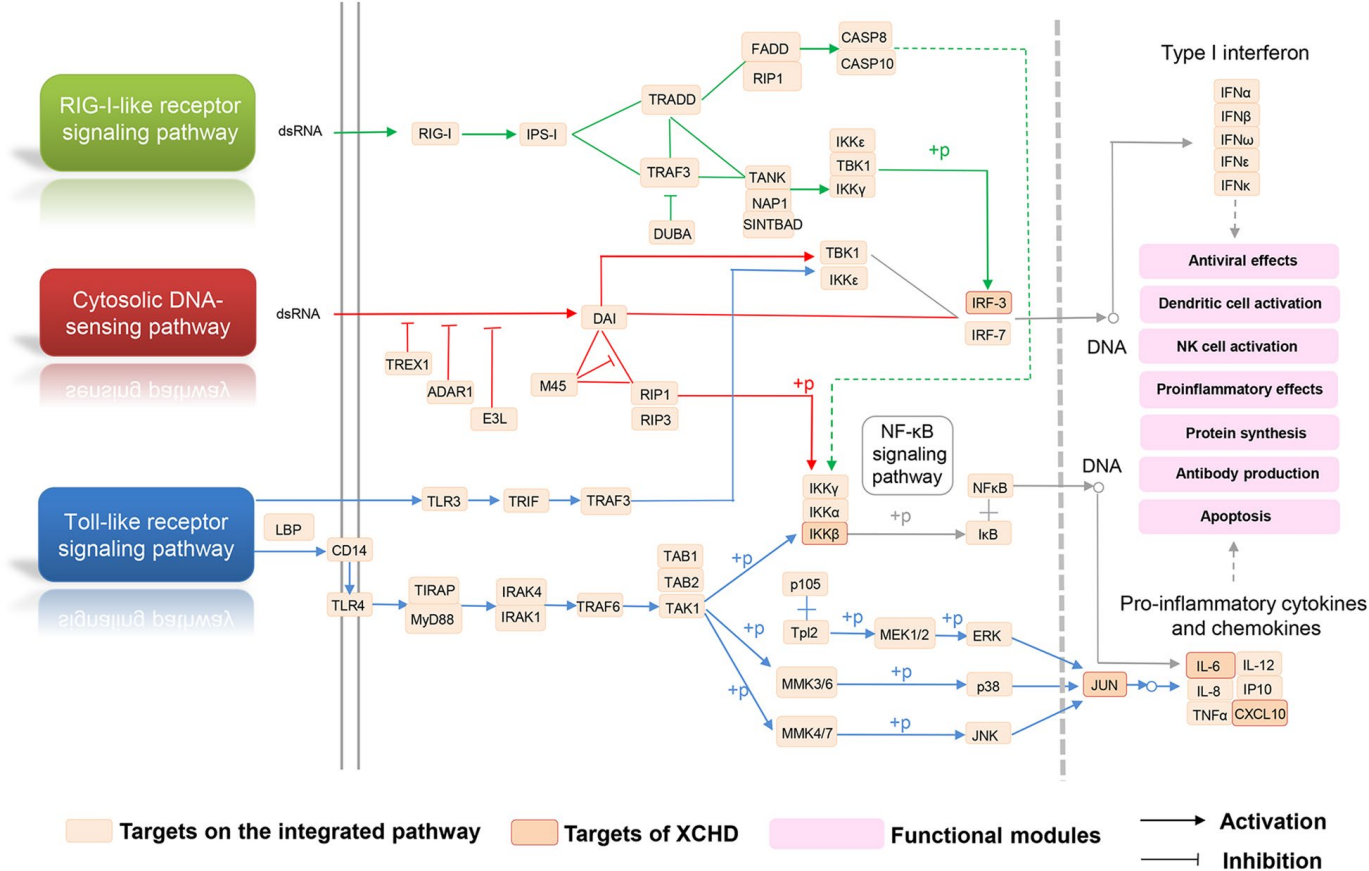
Integrated pathway of XCHD against HCoVs

Since XCHD exerts anti-HCoVs effects through regulating immunity-related pathways. To further illustrate the potential mechanisms of XCHD against CoVs, we constructed an integration focusing on three pathways, RIG-I-like receptor signaling pathway, cytosolic DNA-sensing pathway, and TLR signaling pathway (Fig. 6).

Upon MERS-CoV infection, RIG-I-like receptor signaling pathway could be activated to trigger the inflammatory cascade [44, 45]. The compounds in XCHD could target to several key proteins in the RIG-I-like receptor signaling pathway by regulating several genes expression, including such as IRF3, IKBKB and CXCL10. Cytosolic DNA-sensing pathway is related to the expression of type I IFNs and proinflammatory cytokines [46–48]. As exhibited in Fig. 6, compounds in XCHD act on multiple targets that are involved in cytosolic DNA-sensing pathway, including IL6, IRF3, IKBKB, and CXCL10, which suggested the potential critical role of XCHD in cytosolic DNA-sensing module. TLR signaling pathway provides strong defense against SARS-CoV infection by the myeloid differentiation factor 88 (MyD88) [49–51]. Many compounds in XCHD were shown to target the key genes that participate in TLR signaling pathway, including IRF3, IL6 and JUN, illustrating that XCHD could trigger a broad array of cytokines and chemokines production through TLR signaling pathway.

In a summary, IRF3, IKBKB, JUN, IL6, and CXCL10 were the HCoVs-associated targets regulated by XCHD. These genes were enriched in the three pathways from above, which might be considered as the XCHD-regulated targets to against HCoVs. IRF3, IKBKB, and CXCL10 were present in all three pathways. IL-6 was exhibited in both cytosolic DNA-sensing and TLR signaling pathways. JUN, also known AP1, was shown only in TLR signaling pathway.

### In silico identification of anti-HCoV activity of ingredients from XCHD

We next utilized in silico approaches to precisely identify the active components of XCHD which can exert anti-HCoV effect. According to the systems pharmacology analysis, we identified 161 compounds in XCHD that regulated 37 HCoVs-associated targets. We further utilized a machine learning-based model to assess the drug-likeness of those 161 compounds. The predictive model was established by random forest algorithm and the chemical structure was described by MACCS fingerprint [37]. Using this model, we screened out previously reported compounds with antiviral functions. By comprehensively considering of the chemical structure, content, availability and accessibility, we finally highlighted 30 potential anti-HCoVs compound candidates in XCHD (Supplementary Table S7).

### In vitro validation of the anti-HCoV activity of XCHD

We conducted the experiments to validate whether the 30 compounds screened out from in silico analysis are able to display anti-HCoV activity in vitro. As shown in Table 4, according to CPE assay, 16 compounds out of the 30 tested compounds (hit rate = 53.4%) had inhibitory activity against HCoV-229E virus with selection index (SI) value > 1. Compared to positive control ribavirin (SI > 49.44 ± 19.59), 5 compounds showed a satisfying anti-HCoV activity in Huh7 cells (SI > 5), which are betulinic acid derived from Dazao (SI = 46.77), chrysin derived from Huangqin (SI = 7.49), isoliquiritigenin derived from Chaihu. and Zhigancao (SI = 6.98), schisandrin B (SI = 6.7) and (20R)-Ginsenoside Rh1 (SI > 5.20) derived from Renshen. Together, these data indicated that the 5 compounds above could be the active ingredients in XCHD against HCoV.

**Table 4:** The activity evaluation of constituents from XCHD in HCoV-229E induced CPE reduction assay

Compound	Pubchem ID	TC_50_^a^	IC_50_^b^	SI^c^	Source	Structure
Isoliquiritigenin	638278	12.93	1.85	6.98	Chaihu; Zhigancao	
Eleutheroside E	71312557	> 50	50	> 1.0	Renshen	
Ginsenoside Rb1	9898279	> 50	50	> 1.0	Renshen	
Kaempferide	5281666	28.87	16.67	1.73	Chaihu; Renshen	
Citric acid	311	> 50	50	> 1.0	Chaihu; Renshen; Dazao	
Citropten	2775	28.87	16.67	1.73	Chaihu; Shengjiang	
6-Shogaol	5281794	3.21	1.44	2.23	Banxia; Shengjiang	
( +)-Catechin Hydrate	107957	> 50	38.8	> 1.29	Dazao	
Betulinic acid	64,971	16.67	0.36	46.77	Dazao	
(20R)-Ginsenoside Rh1	21599923	> 50	9.62	> 5.20	Renshen	
Methyl salicylate	4133	> 50	34.67	> 1.44	Chaihu	
Scopoletin	5280460	> 50	50	> 1.0	Chaihu; Dazao	
Chrysin	5281607	34.67	4.63	7.49	Huangqin	
Schisandrin B	108130	28.87	4.31	6.7	Renshen	
Ginsenoside Rg3	9918693	> 50	16.67	> 3.0	Renshen	
L-(-)-Fucose	3034656	> 50	28.87	> 1.73	Renshen	
Paeonol	11092	> 50	50	> 1.0	Banxia; Renshen	
Ribavirin^d^	37542	> 100	2.2 ± 0.87	> 49.44 ± 19.59	-	

^a^TC_50_: 50% cytotoxic concentration (μg/ml)

^b^IC_50_: 50% effective concentration (μg/ml)

^c^SI: Selection index, TC_50_/IC_50_

^d^Positive control drug

### Synergistic effects investigation of the five active compounds in XCHD through compound-target subnetwork

**Fig. 7:**
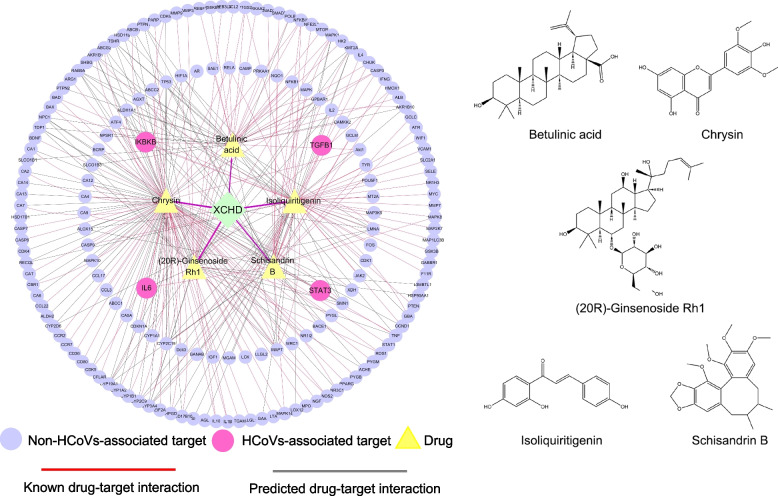
The C-T subnetwork and chemical structures of five active compounds in XCHD against HCoVs

As shown in the in vitro experiments, betulinic acid, chrysin, isoliquiritigenin, schisandrin B, and (20R)-Ginsenoside Rh1 are the most promising anti-HCoVs components of XCHD. To investigate whether they can perform synergistic effects against HCoVs, we established a C-T subnetwork for the five compounds through extracting the corresponding CTIs from the global H-C-T network. As shown in Fig. 7, there were 175 known CTIs and 133 predicted CTIs. Five compounds were connected to 174 target proteins. The chrysin, isoliquiritigenin, betulinic acid, schisandrin B, and (20R)-Ginsenoside Rh1 exhibited degree of 98, 60, 39, 38, and 33, respectively. Furthermore, five proteins, including CYP3A4, LMNA, MAPT, RAB9A, and SMN1 were simultaneously targeted by all the 5 compounds, suggesting that XCHD may exert its synergistic anti-HCoVs effect through regulating these targets. Remarkably, the 5 compounds in XCHD act on the 4 key HCoVs-associated targets including IL6, STAT3, IKBKB, and TGFB1, indicating a multi-target anti-HCoVs mechanism of XCHD. Collectively, XCHD might target the shared genes regulated by these compounds to perform synergistic function and affect 4 key molecules to exert the anti-HCoV activity.

### The conformational affinity assessment of key molecules and HCoVs-associated targets by Libdock

The X-ray crystal structures of IKBKB, IL6, STAT3 and TGFB1 were downloaded using PDB IDs for 3BRT, 1ALU, 6NJS and 4KV5, respectively.

The Libdock results of chrysin, isoliquiritigenin, betulinic acid, schisandrin B, and (20R)-Ginsenoside Rh1 with their respective key targets are shown in the Table 5. When docked with IKBKB, the LibDock scores of betulinic acid and isoliquiritigenin were 123.317 and 101.826, which are similar to the values of ribavirin (reference compound). (20R)-Ginsenoside Rh1 leads the list with a high LibDock value of 110.3, which is better than the reference compounds namely ribavirin (LibDock score, 85.4208) when docked with IL6. Schisandrin B also showed good affinities for STAT3 and TGFB1, with the LibDock score of 102 and 53.5814, while the values of positive control ribavirin were 100.454 and 83.9502.

**Table 5:** The Libdock results of 4 HCoVs-associated targets and 5 key molecules from XCHD

Target Gene Symbol	Compound	Libdock Score
IKBKB	Betulinic acid	123.317
	Isoliquiritigenin	101.826
	Ribavirin	122.055
IL6	Chrysin	77.7398
	Schisandrin B	65.3883
	(20R)-Ginsenoside Rh1	110.3
	Ribavirin	85.4208
STAT3	Schisandrin B	102
	Ribavirin	100.454
TGFB1	Schisandrin B	53.5814
	Ribavirin	83.9502

## Discussion

Currently, the pandemic of COVID-19 still prevails around the world. The shortage of medicine and mutation of HCoV prompt more candidates from a broader scope to be discovered. In this tough campaign against COVID-19, TCM has been widely applied for treating over 85% infected patients and displays a promising effect in China [52]. Some TCM formulas, including XCHD, have been promoted to recommended prescriptions by the *COVID-19 diagnosis and treatment scheme* in China. This gave an inspairation that TCM-derived natural products have strong prospect to be developed into the novel drugs. However, how to identify the active components and elucidate the underlying mechanism of their actions remain challenging, which hinders the further clinical translation and application.

In this study, we provided a systems pharmacology-based framework to identify the potential active components in XCHD and explore the potential anti-HCoVs mechanism. This framework was based on a global H-C-T network, which incorporated 2,454 herb-compound pairs, 90 high-quality HCoVs-associated targets, 45,624 known and predicted compound-target interactions obtained from multiple authoritative databases, predictive network model, and published literatures.

According to the mechanisms of marketed agents against HCoVs (mainly SARS-CoV-2), there are several therapeutic adoption pathways for HCoVs, including the inhibition of viral proteins (3C-like protease and RNA-dependent RNA polymerase), the blockage the spike-ACE2 interaction, and the antagonization of Janus kinase and cytokines [53]. In this study, multi-level systems pharmacology analysis revealed that XCHD-affected HCoV-associated targets may inhibit the entry, transport, and genome replication of virus and may be involved in other biological processes related to innate and adaptive immune response such as TLR signaling pathway, RIG-I-like receptor signaling pathway, cytoplasmic DNA sensing pathway, and IL-6/STAT3 pro-inflammatory signal transduction axis. TLR signaling pathway, RIG-I-like receptor, and cytoplasmic DNA sensing pathway have been well demonstrated to be highly relevant to anti-virus functions. Upon viral infection, RIG-I-like receptors can detect the presence of virus-associated molecular patterns [44] and trigger the activation of type I interferons (IFN) and inflammatory mediators to eliminate the viral pathogens and infected cells. Previous studies implied that activation of RIG-I-like receptor signaling pathway contributes to inflammatory cascade in MERS-CoV-infected macrophages [45]. DNA-dependent activator of IFN regulatory factors (DAI), a cytosolic DNA sensor, are one of the recently described pattern recognition receptors (PRRs). DAI can activate the IRF3- and/or NF-κB-responsive genes, and induces the expression of type I IFNs and proinflammatory cytokines [46–48]. And TLR signaling pathway proceeds from two pathways: the TRIF-mediated pathway induced by Toll-interleukin-1 receptor (TIR)-domain-containing adaptor, and the MyD88-mediated pathway. The latter one activates NF-κB and induces the inflammatory reactions [49]. The importance of TLR signaling pathway on controlling the progression of respiratory virus infections is highly dependent on TRIF and MyD88 [50]. There is evidence that MyD88-independent signaling via the TRIF adaptor protein exerts powerful defense against SARS-CoV infection [51]. IL-6/STAT3 pathway is involved in a non-specific and acute response of innate immune system to pathogen infection [54]. Previous report has revealed that STAT3 was able to inhibit SARS-CoV-2 by regulating ACE2 expression [55].

We further proposed an integrated pathway to comprehensively understand the anti-HCoVs effects of XCHD in different directions and signaling pathways. Meanwhile, with the combination of in silico prediction and in vitro validation, we for the first time discovered 16 compounds in XCHD exerting inhibitory activity against HCoV-229E virus. Among them, betulinic acid, chrysin, isoliquiritigenin, schisandrin B, and (20R)-Ginsenoside Rh1 showed comparable activity as anti-virus drug ribavirin. It was further confirmed that 5 key compounds showed good affinities for the four HCoV-associated targets (IKBKB, IL6, STAT3 and TGFB1) by LibDock. Betulinic acid, chrysin and isoliquiritigenin were detected from Dazao, Huangqin, and Zhigancao, with the content of 1602.008 μg/g, 36.23 ± 8.48 mg/g, and 0.281 ± 0.008 mg/g, respectively [56–58]. Schisandrin B and (20R)-Ginsenoside Rh1 are both compounds contained in Renshen. There was 1.17 mg/ml (20R)-Ginsenoside Rh1 can be obtained from 10% (w/v) ginseng root extract [59]. Betulinic acid was previously tested to inhibit various viruses such as Zika virus, dengue virus, influenza virus and human immunodeficiency virus (HIV) [60–62]. Chrysin was found to fight against influenza virus, herpes-virus and enterovirus 71 [63–65]. Isoliquiritigenin was proved to reduce morbidity and lung inflammation in mice model after influenza virus infection [66]. Schisandrin B was identified as new scaffold of HIV-1 RT inhibitors [67]. (20R)-Ginsenoside Rh1 has been reported to eliminate the cytoprotective phenotype of HIV-1-transduced human macrophages [68]. These evidences indicated that these compounds have effects against various viruses. And their anti-HCoVs efficacy will be verified by further experiments in the near future.

The systems pharmacology-based infrastructure has several progresses: (i) constructing a global H-C-T network by integrating high quality HCoVs associated proteins and comprehensive validated CTIs. The completeness of the H-C-T network was further improved via adding computationally predicted targets inferred by bSDTNBI method; (ii) the systems pharmacology approach was independent from high-quality negative samples or 3D structures of targets, which are critical for algorithms such as molecular docking and machine learning [69]; (iii) considering the strict experimental operating conditions and limited research resources required for SARS-CoV-2 study, the current framework provides a low-threshold but high efficiency in silico approach for drug development.

However, several potential limitations should be addressed in the study. First, although the C-T network was assembled by the large-scale CTIs from accessible databases and computational data via bSDTNBI method, there are still incompleteness to some extend. The drug-induced transcriptome and proteome data should be considered to update into the current C-T network. Second, due to the complexity of the intrinsic interactions and the diversity of components in the mixture of multiple herbs, the current system pharmacology analysis could not accurately reflect the actual biological effects of XCHD on HCoV inhibition in patients [70]. Last, although we have discovered several compounds in XCHD exerting the promising anti-HCoV potential through in vitro HCoV-229E virus-induced cytopathic effect assay, the limited experimental validation of the results by systems pharmacology analysis should be acknowledged. The verification of the in-depth molecular mechanisms, the broader antiviral activity, and the specific therapeutical effect should be further validated by in vivo assays and randomized controlled clinical trials.

## Conclusions

Overall, the systems pharmacology is a booming research field, which can be used to find the potential acting target for traditional medicine. Our study proposed several anti-HCoVs drug candidates from XCHD, which are betulinic acid, chrysin, isoliquiritigenin, schisandrin B, and (20R)-Ginsenoside Rh1. In addition, our analysis show the implicated mechanism of XCHD against HcoVs, including TLR signaling pathway, RIG-I-like receptor signaling pathway, cytoplasmic DNA sensing pathway, and IL-6/STAT3 pro-inflammatory signal transduction axis. All these results shed light on that system pharmacology is a powerful tool to dissect the mechanisms of TCM to cure disease.

## Supplementary Information

**Additional file 1: Supplementary table 1.**

**Additional file 2: Supplementary table 2. **

**Additional file 3: Supplementary table 3. **

**Additional file 4: Supplementary table 4. **

**Additional file 5: Supplementary table 5. **

**Additional file 6: Supplementary table 6. **

**Additional file 7: Supplementary table 7. **

## Data Availability

The original contributions presented in the study are included in the article/supplementary material; further inquiries can be directed to the corresponding authors upon reasonable request.

## Abbreviations

bSDTNBI: Balanced substructure-drug-target network-based inference
CoV: Coronavirus
COVID-19: Coronavirus disease 2019
CPE: Cytopathic effect
CTIs: Compound-target interactions
DAI: IFN regulatory factors
HCoVs: Human coronavirus
H-C-T: Herb-compound-target
IC50: Half inhibitory concentration
LQC: Lianhua Qingwen Capsule
MERS-CoV: Middle East respiratory syndrome coronavirus
PPI: Protein–protein interaction
PRR: Pattern recognition receptors
QFPDD: Qingfeipaidu decoction
SARS-CoV: Severe acute respiratory syndrome coronavirus
SARS-CoV-2: Severe acute respiratory syndrome coronavirus 2
TC50: Half toxic concentration
TCM: Traditional Chinese medicine
XCHD: Xiaochaihu decoction
